# Supplementary material for: Analysis of PPARγ Signaling Activity in Psoriasis
Source: Int J Mol Sci. 2021 Aug 10;22(16):8603. doi: 10.3390/ijms22168603 (PMC8395241; doi:10.3390/ijms22168603)
Supplement: Supplementary file 1 [file ijms-22-08603-s001.zip › Supplemental materials_Analysis of PPARg signaling activity in psoriasis/Pathway models/Models images and html files/Model of PPARG related pathways in psoriasis (short version)/88092.html]

TIRAP


# Protein TIRAP

|  |  |
| --- | --- |
| URN | urn:agi-llid:114609 |
| Total Entities | 0 |
| Connectivity | 418 |
| Name | TIRAP |
| Description | toll-interleukin 1 receptor (TIR) domain containing adaptor protein |
| Notes | The innate immune system recognizes microbial pathogens through Toll-like receptors (TLRs), which identify pathogen-associated molecular patterns. Different TLRs recognize different pathogen-associated molecular patterns and all TLRs have a Toll-interleukin 1 receptor (TIR) domain, which is responsible for signal transduction. The protein encoded by this gene is a TIR adaptor protein involved in the TLR4 signaling pathway of the immune system. It activates NF-kappa-B, MAPK1, MAPK3 and JNK, which then results in cytokine secretion and the inflammatory response. Alternative splicing of this gene results in several transcript variants; however, not all variants have been fully described. [provided by RefSeq, Jul 2008] |
| Primary Cell Localization | Cytoplasm |
| ObjectType | Protein |

---

|  |  |
| --- | --- |
| Pathway | I-cell: CCK-8 Secretion and Regulation of Eating Behavior |
|  | Toll-like Receptors Act through MYD88-TIRAP Signaling |
|  | TLR4 Signaling in Leukocytes |
|  | Toll-like Receptors in Sterile Inflammation |
|  | CD16/CD14 Proinflammatory Monocyte Activation |
|  | AHR Signaling in M1 Macrophages Function |
|  | AXL Receptor Inhibits Macrophages and Dendritic Cells Function |
|  | Adipokines Production by Adipocyte |
|  | Dendritic Cells Function in Atherosclerosis |
|  | RAGE/AGER and S100 Proteins in Cardiovascular Injury |
|  | Inflammatory Reaction in Acne Vulgaris |
|  | Dendritic Cells Function in Psoriasis |
|  | Dendritic Cell Activation in Systemic Lupus Erythematosis |
|  | Plasmacytoid Dendritic Cell in Diabetes Mellitus Type 1 |
|  | TLR2 Signaling in Treg-Cell in Type 1 Diabetes (Animal Model) |
|  | Toll-like Receptors in beta-Cell |
|  | Conventional Dendritic Cell Role in Diabetes Mellitus Type 1 |
|  | Insulin Resistance in Myocytes Induced by Obesity |
|  | Hepatic Stellate Cells in non-Alcoholic Fatty Liver Disease |
|  | Adipokines Production by Adipocyte Impaired in Obesity |
|  | Celiac Disease |
|  | Crohn's Disease |
|  | Dendritic Cell Dysfunction in Crohn's disease |
|  | Dendritic Cell Function in Ulcerative Colitis |
|  | Nicotine anti-Inflammatory Effect in Ulcerative Colitis |
|  | B-Cell Chronic Lymphocytic Leukemia |
|  | Diffuse Large-B-Cell Lymphoma |
|  | Diffuse Large-B-Cell Lymphoma ABC Subtype |
|  | Club-Cell Role in Asthma |
|  | Antigen-Presenting Cell Role in Asthma |
|  | Mucin Production in Goblet Airway Epithelial Cells |
|  | CFTR Facilitates Oxidative Stress in Airway Epithelium in CF |
|  | Airway Epithelial Cell Dysfunction |
|  | Monocytes Function in Gout |
|  | Dendritic Cell Dysfunction in Psoriatic Arthritis |
|  | TLR2 Induced Synovial Fibroblast Activation in Rheumatoid Arthritis |
|  | Toll-like Receptors (TLR) in Periodontitis |
|  | IL1 Receptor Antagonist (IL1RN) Deficiency (DIRA) |
|  | PAPA Syndrome |
|  | TNF Receptor Associated Periodic Syndrome |
|  | Mevalonate Kinase Deficiency |
|  | Blau Syndrome |
|  | IL36 Receptor Antagonist (IL1F5) Deficiency (DITRA) |
|  | Cryopyrin (NLPR3) Associated Periodic Syndromes |
|  | Familial Cold Autoinflammatory Syndrome 2 |
|  | Familial Mediterranean Fever |
|  | Majeed Syndrome |
|  | Gluten Impact on Neuronal System (Hypothesis) |
|  | HMGB1 and IL1B in Neuroinflammation |
|  | HMGB1 and IL1B in Neuronal Hyperexcitation |
|  | Cholesteatoma |
|  | HPV E6 and E7 Induced Disruption of TNFR/NF-kB Signaling in Keratinocytes |
|  | MUC5AC Expression by NTHi and S. Pneumoniae |
|  | Immune System Role in Otitis Media |
|  | Alveolar Macrophage Negative Regulation Declined in COPD |
|  | Alveolar Macrophages Dysfunction in COPD |
|  | T-Cell Cytotoxic Activation |
|  | Alveolar Epithelial Cell Dysfunction in COPD |
|  | Mucin Hyperproduction in Goblet and Mucous Cells |
|  | Toll-like Receptors in Pyelonephritis |
|  | NFKB Canonical Signaling Activation in Cancer |
|  | NFKB Signaling Activation by Blocking of Tumor Suppressors |
|  | NF-kB Canonical Signaling |
|  | TLR4 -> AP-1 Expression Targets |
|  | TLR4 -> AP-1/EGR1/HIF1A Expression Targets |
|  | TLR4 -> NF-kB/IRF Expression Targets |
|  | TLR4 -> IRF Signaling |
|  | TLR4/5/7/9 -> NF-kB Signaling |
|  | TLR -> AP-1 Signaling |
|  | pathway 4 |
|  | Model of PPARG signaling in psoriasis |
|  | Model of PPARG related pathways in psoriasis (short version) |
|  | sars-cov no binding |
|  | AGER feed forward loop in innate immunity |
|  | Dendritic Cell Activation in COVID19 |
|  | 1\_Inflammation in keratinocytes in the pathology of acne |
|  | 1\_The role of Toll-like receptors in pyelonephritis |
|  | 2\_Pathogens stimulate mucins expression in the middle ear |
|  | 3\_Arterial wall inflammation and calcification in atherosclerosis |
|  | 1\_Cigarette smoke causes dysfunction and production of inflammatory mediators in alveolar epithelial cells |
|  | 3\_Role of alveolar macrophages in pulmonary tissue destruction in COPD |
|  | 1\_Th2 cell response in asthma |
|  | 3\_1\_Mucus accumulation in asthma |
|  | 4\_Mucin hyperproduction in goblet and mucous cells in COPD |
|  | 2\_Paneth cell dysfunction in Crohn's disease |
|  | 1\_Defects in response to pathogens in the gut promotes inflammation in Crohn's disease |
|  | 2\_Intestine epithelial cells drive immunological responses in ulcerative colitis |
|  | 1\_Intestine epithelial cell dysfunction in ulcerative colitis |
|  | 2\_Lipotoxicity in NAFLD |
|  | 1\_CD4+ T cell and epithelial cell response in celiac disease |
|  | 5\_Human papilloma virus E6 and E7 proteins promote epithelial cell proliferation |
|  | 4\_3\_Insulin resistance: FFA-related insulin resistance |
|  | 1\_Antigen-presenting cells promote islet dysfunction and immune system activation in T1D |
|  | 2\_Neuron and Schwann cell death |
|  | Figure 4\_Diabetes mellitus Type 1: conventional dendritic cell |

---

|  |  |
| --- | --- |
| Group | other partners for SARS |

---

|  |  |
| --- | --- |
| MedScan ID | 114609 |

---

|  |  |
| --- | --- |
| LocusLink ID | 114609 |
|  | 117149 |
|  | 680127 |

---

|  |  |
| --- | --- |
| Alias | C130027E04 |
|  | adapter Mal |
|  | FLJ42305 |
|  | Tlr4ap |
|  | wyatt |
|  | adaptor protein Wyatt |
|  | BACTS1 |
|  | toll-like receptor 4 adaptor protein |
|  | MAL/TIRAP |
|  | C130027E04Rik |
|  | Toll/IL-1 receptor-associated protein |
|  | Toll/IL1 receptor associated protein |
|  | MyD88-2 |
|  | TIRAP/MAL |
|  | TIR domain containing adaptor protein |
|  | OTTHUMP00000179130 |
|  | LOC681662 |
|  | LOC115469 |
|  | AA407980 |
|  | RIKEN cDNA C130027E04 gene |
|  | Toll/IL-1 receptor associated protein |
|  | Toll/IL1 receptor-associated protein |
|  | TIRAP |
|  | OTTHUMP00000179132 |
|  | Mal |
|  | MyD88 adapter-like protein |
|  | Toll-like receptor adaptor protein |
|  | adapter protein wyatt |
|  | toll-interleukin 1 receptor (TIR) domain containing adaptor protein |
|  | toll/interleukin-1 receptor domain-containing adapter protein |
|  | TIR domain-containing adapter protein |
|  | toll-interleukin 1 receptor (TIR) domain-containing adaptor protein |
|  | toll-like receptor 4 adapter protein |

---

|  |  |
| --- | --- |
| GO ID | 0035663 |
|  | 0035662 |
|  | 0005546 |
|  | 0030674 |
|  | 0046982 |
|  | 0042803 |
|  | 0005080 |
|  | 0070935 |
|  | 0002755 |
|  | 0035665 |
|  | 0007250 |
|  | 0007166 |
|  | 0071221 |
|  | 0071223 |
|  | 0050830 |
|  | 0006954 |
|  | 0045087 |
|  | 0030099 |
|  | 0044130 |
|  | 0030890 |
|  | 0070374 |
|  | 0043123 |
|  | 0046330 |
|  | 0051092 |
|  | 2000340 |
|  | 2000343 |
|  | 0032735 |
|  | 0032738 |
|  | 0045410 |
|  | 0032757 |
|  | 0090023 |
|  | 0090073 |
|  | 0034137 |
|  | 0034141 |
|  | 0034145 |
|  | 0032760 |
|  | 0045088 |
|  | 0032648 |
|  | 0032496 |
|  | 0005737 |
|  | 0005829 |
|  | 0030139 |
|  | 0005886 |
|  | 0032587 |
|  | 0007249 |
|  | 0007257 |
|  | 0002376 |
|  | 0070498 |
|  | 0045089 |
|  | 0032658 |
|  | 0009617 |
|  | 0007165 |
|  | 0034146 |
|  | 0016020 |
|  | 0005777 |
|  | 0005622 |
|  | 0034142 |
|  | 0045171 |
|  | 0034134 |
|  | 0038123 |
|  | 0038124 |
|  | 0005654 |
|  | 0002224 |
|  | 0008063 |
|  | 0034130 |
|  | 0072568 |
|  | 0004888 |
|  | 0031224 |
|  | 0032722 |

---

|  |  |
| --- | --- |
| KEGG ID | hsa:114609 |
|  | mmu:117149 |
|  | rno:680127 |

---

|  |  |
| --- | --- |
| Organism | Homo sapiens {Organism urn:agi-taxid:9606} |
|  | Mus musculus {Organism urn:agi-taxid:10090} |
|  | Rattus norvegicus {Organism urn:agi-taxid:10116} |
|  | Homo sapiens |
|  | Mus musculus |
|  | Rattus norvegicus |

---

|  |  |
| --- | --- |
| Mouse chromosome position | 9 |

---

|  |  |
| --- | --- |
| OMIM ID | 606252 |
|  | 614382 |
|  | 610799 |
|  | 607948 |
|  | 611162 |

---

|  |  |
| --- | --- |
| Rat chromosome position | 8q21 |

---

|  |  |
| --- | --- |
| Hugo ID | 17192 |
|  | HGNC:17192 |

---

|  |  |
| --- | --- |
| Human chromosome position | 11q24.2 |

---

|  |  |
| --- | --- |
| Swiss-Prot Accession | A0A024R3M4 |
|  | P58753 |
|  | P58753.2 |
|  | H3BKL1 |
|  | Q542S6 |
|  | Q3UB38 |
|  | Q99JY1 |
|  | Q99JY1.1 |
|  | D3ZXA5 |
|  | B3KW65 |
|  | Q56UH9 |
|  | Q56UI0 |
|  | Q8N5E5 |
|  | Q91ZW0 |

---

|  |  |
| --- | --- |
| GenBank ID | NC\_000011 |
|  | NM\_001039661 |
|  | NP\_001034750 |
|  | NM\_001318777 |
|  | NP\_001305706 |
|  | NM\_148910 |
|  | NP\_683708 |
|  | NM\_001318776 |
|  | NP\_001305705 |
|  | NG\_011523 |
|  | AP001318 |
|  | AY282416 |
|  | AAP31973 |
|  | CH471065 |
|  | EAW67687 |
|  | EAW67688 |
|  | EAW67689 |
|  | GU646880 |
|  | AB446477 |
|  | BAG55254 |
|  | AF378129 |
|  | AAL05627 |
|  | AF406652 |
|  | AAL01160 |
|  | AF410783 |
|  | AAL05036 |
|  | AK124298 |
|  | BAG54027 |
|  | AK313147 |
|  | BAG35965 |
|  | AY576785 |
|  | AAT90417 |
|  | AY576786 |
|  | AAT90418 |
|  | AY576787 |
|  | AAT90419 |
|  | BC032474 |
|  | AAH32474 |
|  | P58753 |
|  | NC\_000075 |
|  | NM\_001177845 |
|  | NP\_001171316 |
|  | NM\_001177846 |
|  | NP\_001171317 |
|  | NM\_054096 |
|  | NP\_473437 |
|  | NM\_001177847 |
|  | NP\_001171318 |
|  | AC140448 |
|  | CH466522 |
|  | EDL25378 |
|  | EDL25379 |
|  | EDL25380 |
|  | AF378130 |
|  | AAL05628 |
|  | AF410784 |
|  | AAL05037 |
|  | AK036431 |
|  | BAC29426 |
|  | AK080135 |
|  | BAC37831 |
|  | AK081514 |
|  | AK151117 |
|  | BAE30126 |
|  | AK168494 |
|  | BAE40381 |
|  | BC005584 |
|  | AAH05584 |
|  | BY507516 |
|  | BY740695 |
|  | BY743490 |
|  | Q99JY1 |
|  | NC\_005107 |
|  | XR\_001839296 |
|  | XM\_017596001 |
|  | XP\_017451490 |
|  | XM\_017596002 |
|  | XP\_017451491 |
|  | XM\_001055833 |
|  | XP\_001055833 |
|  | XM\_017596003 |
|  | XP\_017451492 |
|  | XM\_017596000 |
|  | XP\_017451489 |
|  | AC\_000076 |
|  | XR\_001844589 |
|  | XM\_017603603 |
|  | XP\_017459092 |
|  | XM\_017603604 |
|  | XP\_017459093 |
|  | XM\_002727021 |
|  | XP\_002727067 |
|  | XM\_017603605 |
|  | XP\_017459094 |
|  | XM\_017603602 |
|  | XP\_017459091 |
|  | AAHX01054041 |
|  | AAHX01054042 |
|  | AC111781 |
|  | CH474007 |
|  | EDL83279 |
|  | EDL83280 |
|  | XR\_356167 |
|  | AABR06054057 |
|  | XR\_356166 |
|  | DQ894164 |
|  | ABM85090 |
|  | XP\_005271456 |
|  | XR\_428968 |
|  | XP\_006226425 |
|  | DQ890988 |
|  | XM\_005271399 |
|  | XP\_006242856 |
|  | AC\_000031 |
|  | ABBA01031224 |
|  | XM\_006226363 |
|  | XM\_011542576 |
|  | XM\_011542577 |
|  | XM\_011542578 |
|  | XM\_011542579 |
|  | AC\_000143 |
|  | XM\_011542580 |
|  | XM\_011542581 |
|  | NC\_018922 |
|  | XM\_011542582 |
|  | AAHY01077101 |
|  | ABM81914 |
|  | XM\_006242794 |
|  | XP\_011540884 |
|  | XP\_011540881 |
|  | XP\_011540880 |
|  | XP\_011540883 |
|  | XP\_011540882 |
|  | XR\_348402 |
|  | XR\_348403 |
|  | XP\_011540878 |
|  | AMYH02025746 |
|  | XP\_011540879 |
|  | NT\_033899 |
|  | NW\_001838043 |
|  | NW\_925173 |
|  | AC\_000054 |
|  | Q56UH9 |
|  | NT\_039472 |
|  | NW\_001030907 |
|  | Q3UB38 |
|  | Q542S6 |
|  | NW\_001084868 |
|  | NW\_047799 |

---

|  |  |
| --- | --- |
| Swiss-Prot ID | TIRAP\_HUMAN |
|  | TIRAP\_MOUSE |
|  | Q3UB38\_MOUSE |
|  | Q56UH9\_HUMAN |
|  | Q542S6\_MOUSE |

---

|  |  |
| --- | --- |
| Cell Localization | Cytoplasm |
|  | Cell membrane |
|  | Membrane |

---

|  |  |
| --- | --- |
| Ensembl ID | ENSG00000150455 |
|  | ENSP00000376447.2 |
|  | ENST00000392680.6 |
|  | ENSP00000376446.1 |
|  | ENST00000392679.5 |
|  | ENSP00000376445.3 |
|  | ENST00000392678.7 |
|  | ENSMUSG00000032041 |
|  | ENSMUSP00000135435.1 |
|  | ENSMUST00000175765.7 |
|  | ENSMUSP00000135876.1 |
|  | ENSMUST00000176685.7 |
|  | ENSMUSP00000135462.1 |
|  | ENSMUST00000177129.7 |
|  | ENSMUSP00000135224.1 |
|  | ENSMUST00000176531.7 |
|  | ENSRNOG00000021420 |
|  | ENSRNOP00000032025.5 |
|  | ENSRNOT00000038312.5 |
|  | ENSMUSP00000135435 |
|  | ENSMUSP00000135876 |
|  | ENSMUST00000176685 |
|  | ENSP00000436967 |
|  | ENST00000392680 |
|  | ENST00000479770 |
|  | ENSMUSP00000135462 |
|  | ENSMUST00000177129 |
|  | ENSMUST00000176531 |
|  | ENSMUSP00000135224 |
|  | ENSMUST00000175765 |
|  | ENSRNOT00000038312 |
|  | ENST00000392678 |
|  | ENST00000392679 |
|  | ENSP00000376445 |
|  | ENSP00000376446 |
|  | ENSP00000376447 |
|  | ENSRNOP00000032025 |

---

|  |  |
| --- | --- |
| MGI ID | MGI:2152213 |
|  | 2152213 |

---

|  |  |
| --- | --- |
| RGD ID | 1595496 |

---

|  |  |
| --- | --- |
| Unigene ID | Mm.23987 |
|  | Hs.537126 |

---

|  |  |
| --- | --- |
| Homologene ID | 14285 |

---

|  |  |
| --- | --- |
| IPI ID | IPI00104143 |
|  | IPI00171090 |
|  | IPI00718966 |
|  | IPI00320042 |
|  | IPI00606468 |
|  | IPI00370462 |
|  | IPI00388057 |

---

|  |  |
| --- | --- |
| Ensembl Gene ID | ENSG00000150455 |

---
